# Beyond chronological age: a decision tree model integrating nutritional and renal function indicators for risk prediction in pre-frail older adults with hepatolithiasis

**DOI:** 10.3389/fmed.2026.1860761

**Published:** 2026-08-04

**Authors:** Yi Qin, Feiyuan Zeng, Rilian Ou, Yun Huang, Yuan Yu, Zhongying Su

**Affiliations:** 1 The First Affiliated Hospital of Guangxi University of Chinese Medicine, Nanning, Guangxi, China; 2 Faculty of Nursing, Guangxi University of Chinese Medicine, Nanning, Guangxi, China; 3 Faculty of Nursing, Hunan University of Medicine, Huaihua, Hunan, China; 4 Wuzhou Gongren Hospital, Wuzhou, Guangxi, China

**Keywords:** decision tree, hepatolithiasis, older adults, pre-frail, propensity score matching, risk prediction model

## Abstract

**Objectives:**

This study develops a decision tree model to predict risks and identify key factors in pre-frail older adults hepatolithiasis patients and performance of models.

**Methods:**

The study included 451 pre-frail older adults. Two balanced sample groups totaling 294 cases were obtained via Propensity Score Matching (PSM). Risk prediction models for hepatolithiasis were constructed based on the full sample and the two groups via applying the Classification and Regression Tree algorithm. Models’ performance was evaluated by 10-fold cross-validation, ROC, and AUC, and models’ performance and key factors for hepatolithiasis risk were compared and analyzed.

**Results:**

A total of 451 pre-frail older adults were included. PSM (1:1 ratio) was conducted to reduce confounding factors, with 147 matched pairs. In the full-sample decision tree model, age (>66.85 years) was identified as the primary split node, followed sequentially by strongly positive urine protein, marital status (married, widowed), height, and educational level. The model achieved a classification accuracy of 72.4%, with an area under the curve (AUC) of 0.795 (95% CI: 0.744–0.845). Following PSM, two subgroup decision tree models were developed. The Group 1 model identified age (>66.45 years), body mass index (>21.40 kg/m^2^), white blood cell count (>6.33 × 10^9^/L), Scr (>87.00 μmol/L), fasting blood glucose (>5.51 mmol/L), triglycerides (≥1.52 mmol/L), and conjugated bilirubin (>5.15 μmol/L) as key risk factors. This model demonstrated superior discriminative ability with an AUC of 0.914 (95% CI: 0.867–0.960). The Group 2 model identified age (>66.95 years), positive urine protein, conjugated bilirubin (>3.15 μmol/L), waist circumference (>87 cm), blood urea nitrogen (>4.12 mmol/L), and conjugated bilirubin (>3.65 μmol/L), and ALT (>11.75 U/L) as key predictors, achieving an AUC of 0.877 (95% CI: 0.820–0.934). Age was consistently identified as the dominant risk factor across all models, a finding further illustrated by a Sankey diagram and ROC. While both matched models showed robust performance, cross-validation indicated a potential risk of overfitting, likely due to the limited sample size.

**Conclusion:**

Decision tree risk prediction models for hepatolithiasis were developed in pre-frail older adults. Age emerged as the dominant risk factor across all models, with additional predictors involving nutritional, metabolic, and renal function indicators. Propensity score–matched subgroup models demonstrated superior discriminative performance compared to the full-sample model. These findings indicate that decision tree modeling, especially when combined with PSM, provides an interpretable and effective approach for identifying high-risk individuals in this vulnerable population. Nevertheless, the potential for overfitting underscores the necessity of external validation using larger, independent cohorts to ensure model generalizability. Future efforts may integrate ensemble learning algorithms to enhance model stability and stratify populations based on advanced age combined with metabolic or hepatic dysfunction profiles, thereby enabling more targeted prevention and intervention strategies for hepatolithiasis.

## 1 Introduction

With the acceleration of global population aging, older adults health has become a core issue in public health (1). Geriatric syndromes, especially the pre-frail state, are becoming a key focus in geriatric research due to their reversibility and close association with adverse health outcomes (2). Constructing precise and efficient risk prediction models for early identification and stratified intervention of specific diseases is a key strategy for achieving proactive health management and optimizing healthcare resource allocation. Globally, the older adults population continues to grow, with an estimated 2.1 billion people aged 60 and over by 2050 (3). China is one of the fastest-aging countries in the world. By the end of 2022, the population aged 60 and over exceeded 280 million, accounting for 19.8% of the total population (4). Within this vast older adults population, a significant proportion are in a pre-frail state. The prevalence of pre-frailty among community-dwelling older adults in China is 43% (5). This stage represents a crucial intervention window for disease prevention and functional maintenance. However, its pathological and physiological characteristics have also created a unique internal environment that may make individuals more prone to specific age-related diseases, including liver and gallbladder disorders. The persistent low-grade inflammatory state commonly observed in pre-frail individuals can promote biliary stasis by impairing bile duct peristalsis and altering the composition of bile acids. Concurrently, insulin resistance and dyslipidemia, frequent metabolic components of pre-frailty, may facilitate cholesterol supersaturation in bile, a well-established lithogenic factor. Furthermore, age-related decline in immune surveillance, which is accentuated in the pre-frail state, may impair the clearance of bacterial pathogens that ascend into the biliary tract, thereby increasing the risk of recurrent cholangitis and pigment stone formation. Hepatolithiasis are a common hepatobiliary system disease characterized by stone formation within the intrahepatic bile ducts, which can lead to serious complications such as cholangitis, liver abscess, biliary cirrhosis, and even cholangiocarcinoma (6). The incidence of hepatolithiasis varies geographically, with prevalence rates as high as 30% in East Asia (7). Its incidence among the older adults is also increasing (8). Among older adults patients seeking medical attention for biliary symptoms, the proportion is even higher. China is a high-burden country for hepatolithiasis, while diagnosis rates in Western countries have seen a slight increase, attributed to improvements in imaging techniques, increased clinical awareness, and immigration from endemic areas (9). The prolonged and recurrent nature of hepatolithiasis not only causes suffering like recurrent abdominal pain and fever, severely affecting quality of life, but also imposes a heavy caregiving and economic burden on families while consuming substantial social healthcare resources, making it a significant public health problem.

Currently, systematic research on the risk of hepatolithiasis in this specific high-risk group of pre-frail older adults is still insufficient. Existing risk prediction models are mostly based on clinical patients, lacking a community prevention perspective, and rarely incorporate the important health status of pre-frailty as a core predictor. Decision tree models, due to their intuitive rules and ease of interpretation, are well-suited for risk stratification and health guidance in community settings. However, the stability of model results faces challenges in observational data with confounding bias. This study aims to utilize the decision tree algorithm and propensity score matching (PSM) to control for confounding factors, construct a risk prediction model for hepatolithiasis in pre-frail older adults, explore differences in model characteristics under different data processing strategies, evaluate model performance and stability, and explore key risk factors, providing empirical evidence for risk stratification, precise intervention, and establishing robust community screening tools and intervention grouping schemes for hepatolithiasis in pre-frail older adults.

## 2 Materials and methods

### 2.1 Study design

This study is a cross-sectional survey.

### 2.2 Study participants

Using purposive sampling, 152 pre-frail older adults diagnosed with hepatolithiasis who met inclusion and exclusion criteria were initially selected from the Nanmian Community Health Service Center in Nanning, Guangxi. Since the required sample size for this study was at least 450 pre-frail older adults participants, an additional 299 pre-frail older adults without hepatolithiasis were included, totaling 451 pre-frail older adults in the study. Diagnostic Criteria is the confirmed diagnosis of hepatolithiasis by abdominal ultrasound, computed tomography, or magnetic resonance cholangiopancreatography. Inclusion criteria included: (1) Age ≥ 60 years; (2) Meeting the diagnostic criteria for hepatolithiasis; (3) Meeting the pre-frail criteria assessed by FI (0.08 ≤ FI ≤ 0.25) (10); (4) Clear consciousness and ability to cooperate with the survey and examinations; (5) Voluntary signing of informed consent. Exclusion criteria: included (1) Comorbid severe heart, brain, lung, or kidney failure or advanced malignant tumors; (2) Presence of cognitive impairment or mental illness preventing cooperation; (3) Incomplete data. With the presence of hepatolithiasis as the dependent variable, PSM was performed 1:1 by using SPSS 27.0. Due to the difficulty in collecting pre-frail older adults samples, the caliper value was set to 0.1. A total of 147 matched pairs of pre-frail older adults were obtained, divided into two groups namely Group 1 and Group 2.

### 2.3 Sample size calculation

This study referred to the Kendall principle for sample size calculation in cross-sectional studies (11), where the sample size should be at least 5–10 times the number of independent variables. This study included 36 variables, considering a 20% attrition rate. The final sample size = base sample size/(1–attrition rate), N = 36 × 10/(1–0.2) = 360/0.8 = 450. Therefore, this study planned to include 451 pre-frail older adults.

### 2.4 Research instruments

Research instruments includes general information questionnaire and Frailty Index (FI). General information questionnaire is designed by the researchers based on literature review, including age, marital status, education level, height, weight, body mass index, waist circumference, hypertension, left and right blood pressure, diabetes, smoking, alcohol consumption, dental problems, exercise habits, allergy history, self-care ability assessment, white blood cells, platelets, urine protein, urine glucose, urine ketones, urine occult blood, fasting blood glucose, ECG findings, serum alanine aminotransferase, serum aspartate aminotransferase, total bilirubin, conjugated bilirubin, serum creatinine, blood urea nitrogen, total cholesterol, triglycerides, serum low-density lipoprotein (LDL-C), serum high-density lipoprotein cholesterol (HDL-C), with total 35 items. FI model established by Mitnitski is used to assess frailty by quantifying the accumulation of age-related health deficits (10). The FI in this study was assessed using 32 items, including hypertension, left and right blood pressure, diabetes, smoking, alcohol consumption, dental problems, exercise habits, allergy history, self-care ability assessment (five items), white blood cells, platelets, urine protein, urine glucose, urine ketones, urine occult blood, fasting blood glucose, ECG findings, serum alanine aminotransferase, serum aspartate aminotransferase, total bilirubin, conjugated bilirubin, serum creatinine, blood urea nitrogen, total cholesterol, triglycerides, serum LDL-C, serum HDL-C, with total 32 items. The FI scoring formula is: FI score = sum of health deficit scores/32. Typically, FI ≤ 0.08 is defined as non-frail, 0.08 ≤ FI ≤ 0.25 as pre-frail, and FI ≥ 0.25 as frail (10). All blood and urine samples were uniformly collected at the community health service center and sent to the laboratory for testing.

### 2.5 Data collection time and method

Data were collected at Nanning Community Health Service Center in Nanning from January 2022 to December 2024 via applying purposive sampling. Detailed explanations of the study’s content and procedures were provided to the 451 eligible older adults. The purpose and use of the questionnaire were explained. After obtaining consent, participants voluntarily signed informed consent forms and completed the questionnaire. Researchers carefully checked the questionnaires to ensure completeness and authenticity. Each questionnaire took 5–8 min to complete. Uniformly trained researchers collected general data, blood biochemistry, and urine test data from the participants. Physical examinations, including height, weight, waist circumference, blood pressure, and collection of venous blood and urine specimens, were completed at the community health service center. This study was approved by the Ethics Committee of Guangxi University of Chinese Medicine (Approval No. GXUCMIRBTM2024-02-118). All this research was conducted in accordance with both the Declarations of Helsinki and Istanbul.

### 2.6 Statistical analysis methods

**FIGURE 1:**
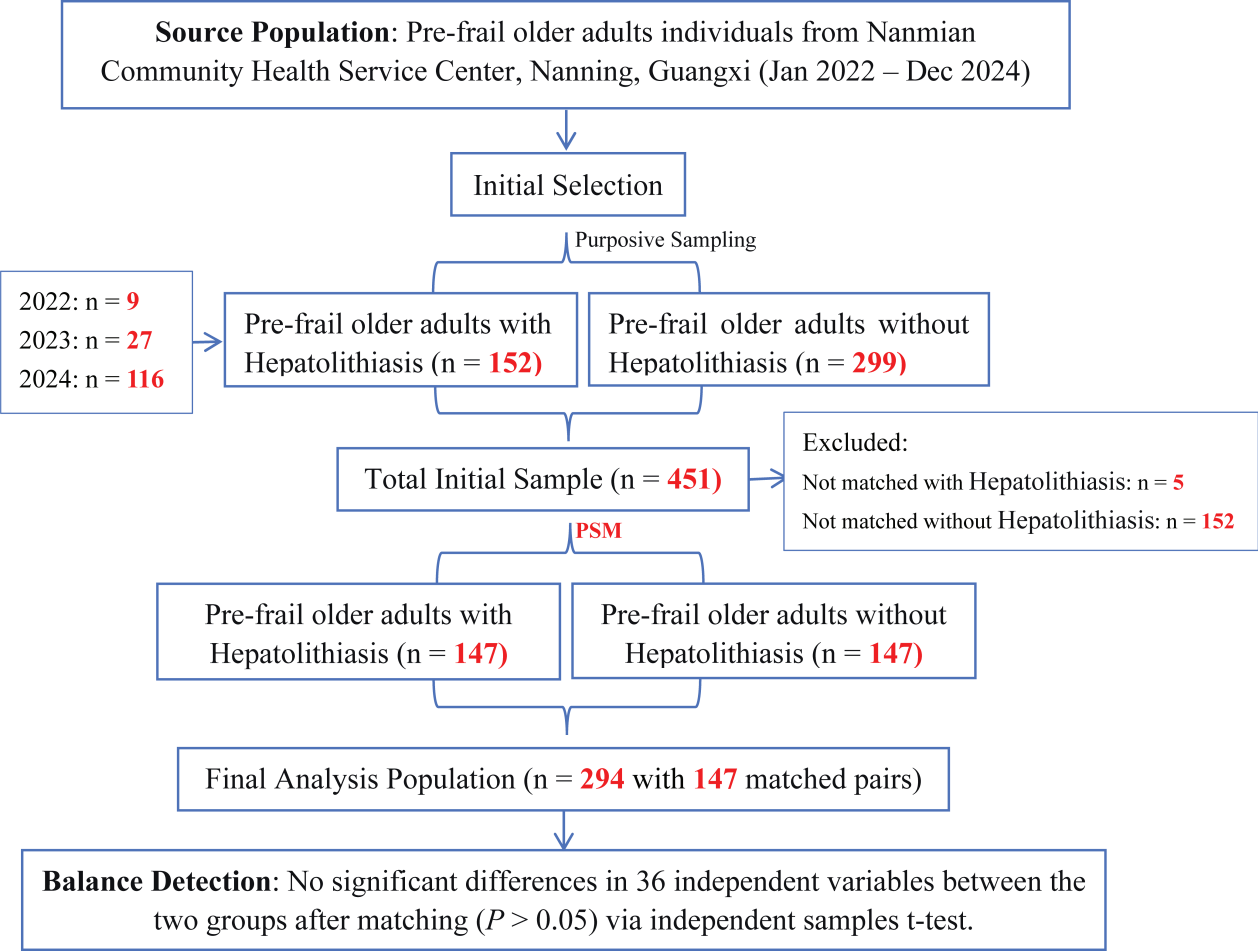
Flow chart of sample inclusion and exclusion.

SPSS 27.0 software was used for data analysis. Continuous variables were expressed as mean ± standard deviation or median and interquartile range for general data analysis; Categorical variables were expressed as frequency and percentage. For constructing the hepatolithiasis risk prediction model, with the presence of hepatolithiasis as the outcome variable, the decision tree algorithm and Gini index were used as the splitting criterion. The optimal pruning was determined through 10-fold cross-validation. A decision tree prediction model for hepatolithiasis risk was constructed for the 294 pre-frail older adults (full-sample model). To construct a more precise risk prediction model, the 294 pre-frail older adults were divided into two groups via PSM to control for baseline confounders. With the presence of hepatolithiasis as the outcome, all independent variables were used as matching variables. Nearest neighbor matching was used with a caliper value of 0.1, with 1:1 of matching by using PSM, resulting in two balanced case-control subsets, each with 147 pre-frail older adults. Group 1 comprised 74 patients with hepatolithiasis and 73 without. Group 2 comprised 73 patients with hepatolithiasis and 74 without. After successful matching, an independent samples *t*-test was used to test for differences in independent variables between the two groups for balance detection. Statistical results showed no significant differences in the 36 independent variables between the two groups after matching (*P* > 0.05). To test the stability of the model in balanced samples, identical decision tree models were independently constructed on the Group 1 and Group 2 data. The full-sample model, Group 1 model, and Group 2 model were compared and analyzed for the selected risk factor nodes and structural differences (Figure 1).

### 2.7 Quality control

Epidemiology and statistics experts were consulted during the study design phase. Investigators were strictly trained and used uniform instructions. All laboratory tests followed standardized procedures and quality control. Data entry was performed by two individuals and checked for completeness and accuracy.

## 3 Results

### 3.1 Prevalence of hepatolithiasis among pre-frail older adults

A total of 152 pre-frail older adults with hepatolithiasis and 299 pre-frail older adults without hepatolithiasis were included in this study, thereinto, 36 (1.23%) pre-frail older adults with hepatolithiasis was selected via applying purposive sampling from 2,907 physical examination pre-frail eldrly in 2022 and in 2023 and 116 (4.18%) pre-frail older adults with hepatolithiasis from 2,773 physical examination pre-frail eldrly in 2024, therefore the prevalence of hepatolihiasis among physical examination pre-frail eldrly in 2022 to 2024 is 2.68%. Total 451 pre-frail older adults with or without hepatolithiasis were recruited in this study. In order to reduced selected bias, PSM was used to reduce confounding factors with matching at a 1:1 ratio, aim to compare the model’s performance. 451 pre-frail older adults were matched as Group 1 (*n* = 147) and Group 2 (n = 147). In Group 1, 74 (50.3%) had hepatolithiasis and 73 (49.7%) had no hepatolithiasis. In Group 2, 73 (49.7%) had hepatolithiasis and 74 (50.3%) had no hepatolithiasis. There were no statistically significant differences in the 36 independent variables between the two groups (*P* > 0.05).

### 3.2 Full-sample decision tree model

**FIGURE 2:**
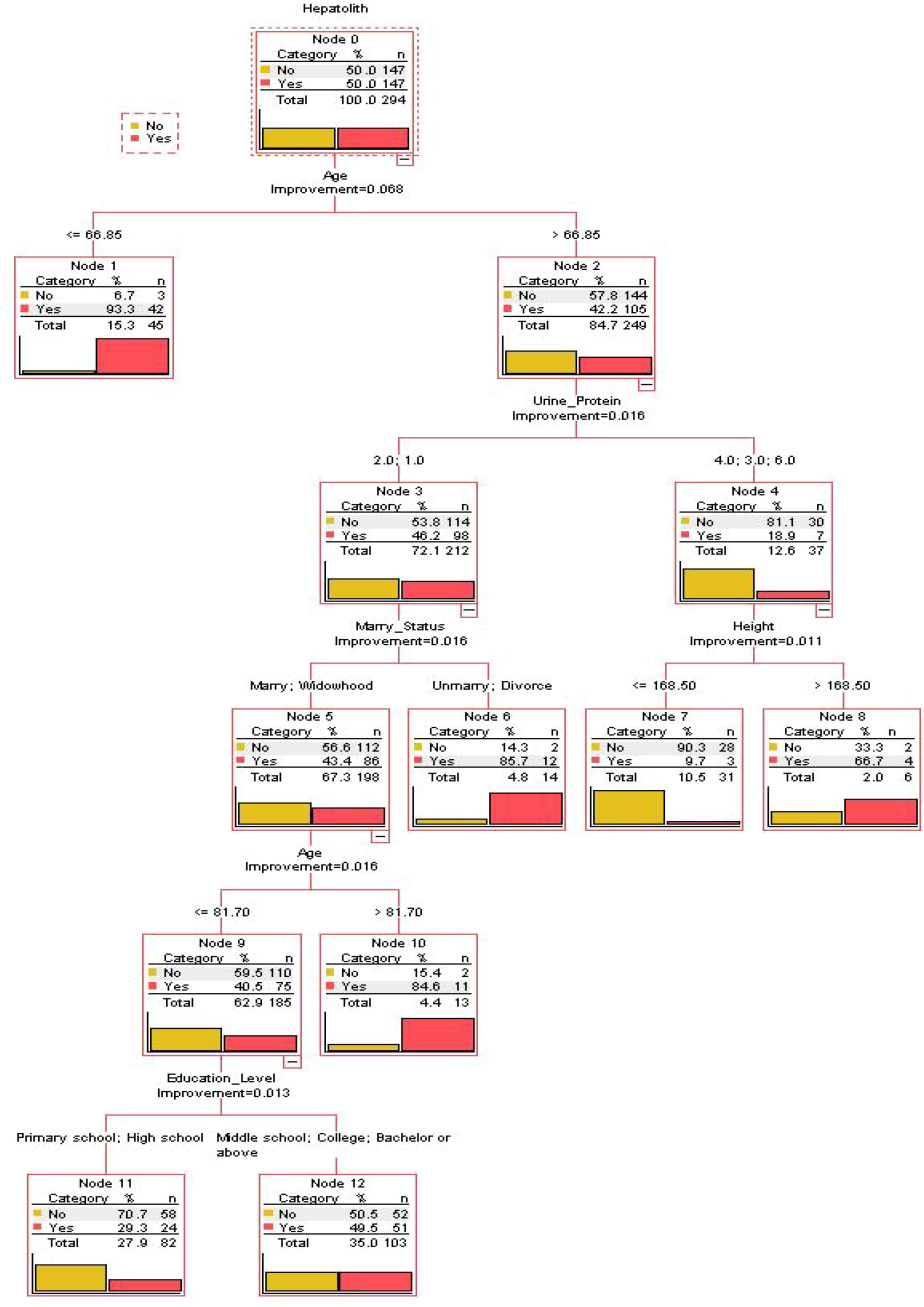
Decision tree model of factors influencing hepatolithiasis among pre-frail older adults for full sample group.

A risk prediction full-sample decision tree model for hepatolithiasis was constructed based on 294 pre-frail older adults by using the PSM-matched total cohort (147 pairs, *n* = 294). The model had 13 nodes, seven terminal nodes, and a maximum tree depth of 5. After 10-fold cross-validation, the model’s classification accuracy in the training set was 70.4%. The prediction accuracy for the non-hepatolithiasis category was 93.9% (138/147), and for the hepatolithiasis category, it was 46.9% (69/147). The model’s resubstitution risk was 0.296, and the cross-validation risk was 0.469, indicating the model had certain robustness but also some risk of overfitting. Gain analysis showed that node 1 (*n* = 45) had the highest identification efficiency for hepatolithiasis patients, with a response rate of 93.3% and an index of 186.7%, being the key node for identifying high-risk individuals. The primary split node was age (>66.85 years), followed sequentially by strongly positive urine protein, marital status (married, widowed), height, and educational level. This indicates that the risk of hepatolithiasis is closely related to age (>66.85 years), strongly positive urine protein, marital status, height, and educational level. The decision tree model effectively integrated these complex relationships, forming interpretable risk stratification pathways (Figure 2).

### 3.3 Comparison of performance of decision tree models after PSM

**FIGURE 3:**
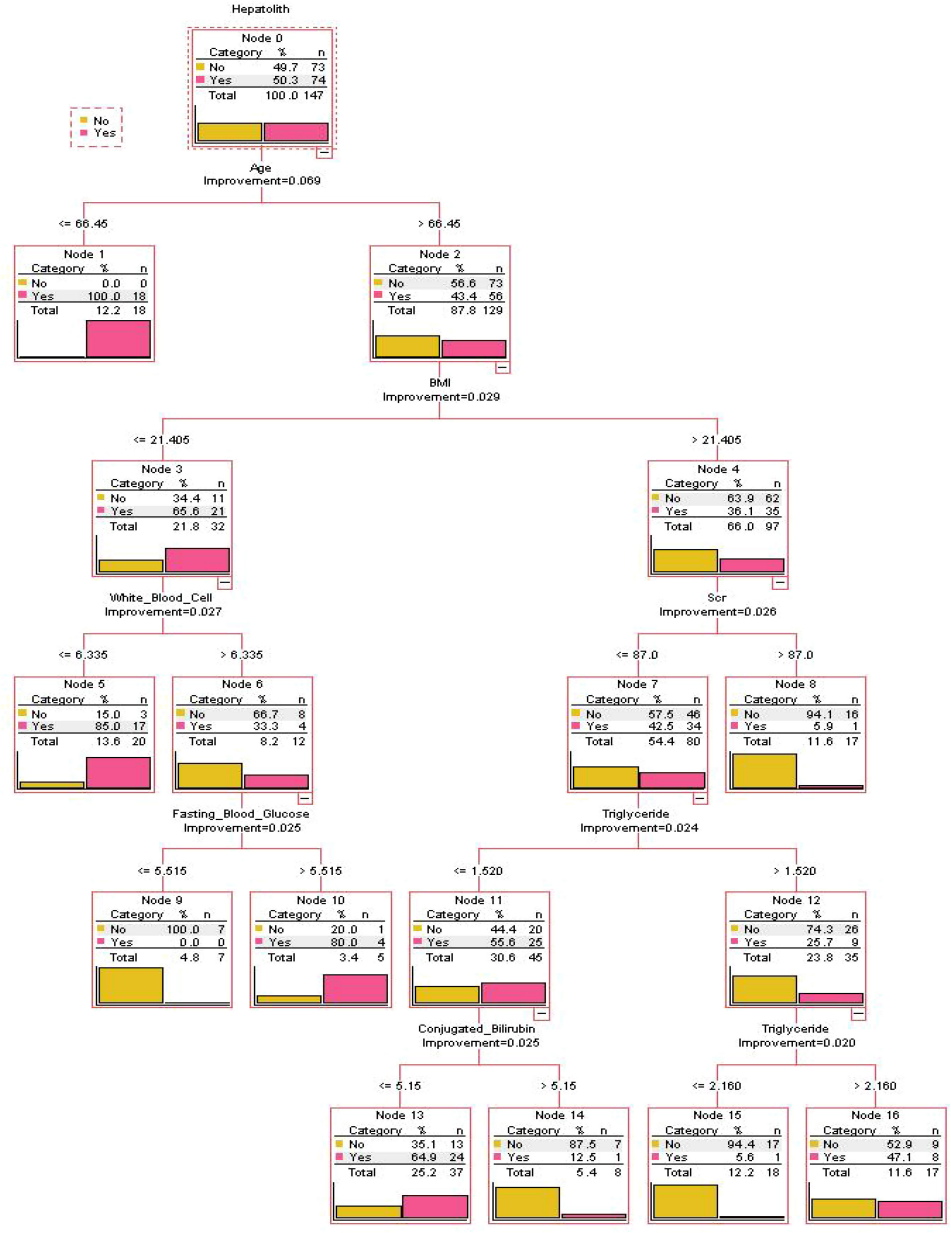
Decision tree model of factors influencing hepatolithiasis among pre-frail older adults for Group 1.

The Group 1 decision tree model had 15 nodes, nine terminal nodes, and a maximum tree depth of five. After 10-fold cross-validation, the model performed excellently in the training set, with an overall classification accuracy of 81.0%. The prediction accuracy for non-hepatolithiasis and hepatolithiasis was 76.7% (56/73) and 85.1% (63/74), respectively. The model’s resubstitution risk was low (0.190), but the cross-validation risk was high (0.544), again suggesting overfitting, possibly related to the relatively small sample size. The gain table showed that node 1 (*n* = 18), node 5 (*n* = 20), and node 10 (*n* = 5) had the highest efficiency in identifying hepatolithiasis patients, with response rates of 100.0%, 85.0%, and 80.0%, respectively. The primary split node was age (>66.45 years), followed sequentially by risk factors for hepatolithiasis including body mass index (>21.40 kg/m^2^), white blood cell count (>6.33 × 10^9^/L), Scr (>87.00 μmol/L), fasting blood glucose (>5.51 mmol/L), triglycerides (≥1.52 mmol/L), and conjugated bilirubin (>5.15 μmol/L). This result, in the matched sample, further confirmed the core role of age (>66.45 years), body mass index (>21.40 kg/m^2^), white blood cell count (>6.33 × 10^9^/L), Scr (>87.00 μmol/L), fasting blood glucose (>5.51 mmol/L), triglycerides (≥1.52 mmol/L), and conjugated bilirubin (>5.15 μmol/L) in hepatolithiasis risk prediction (Figure 3).

**FIGURE 4:**
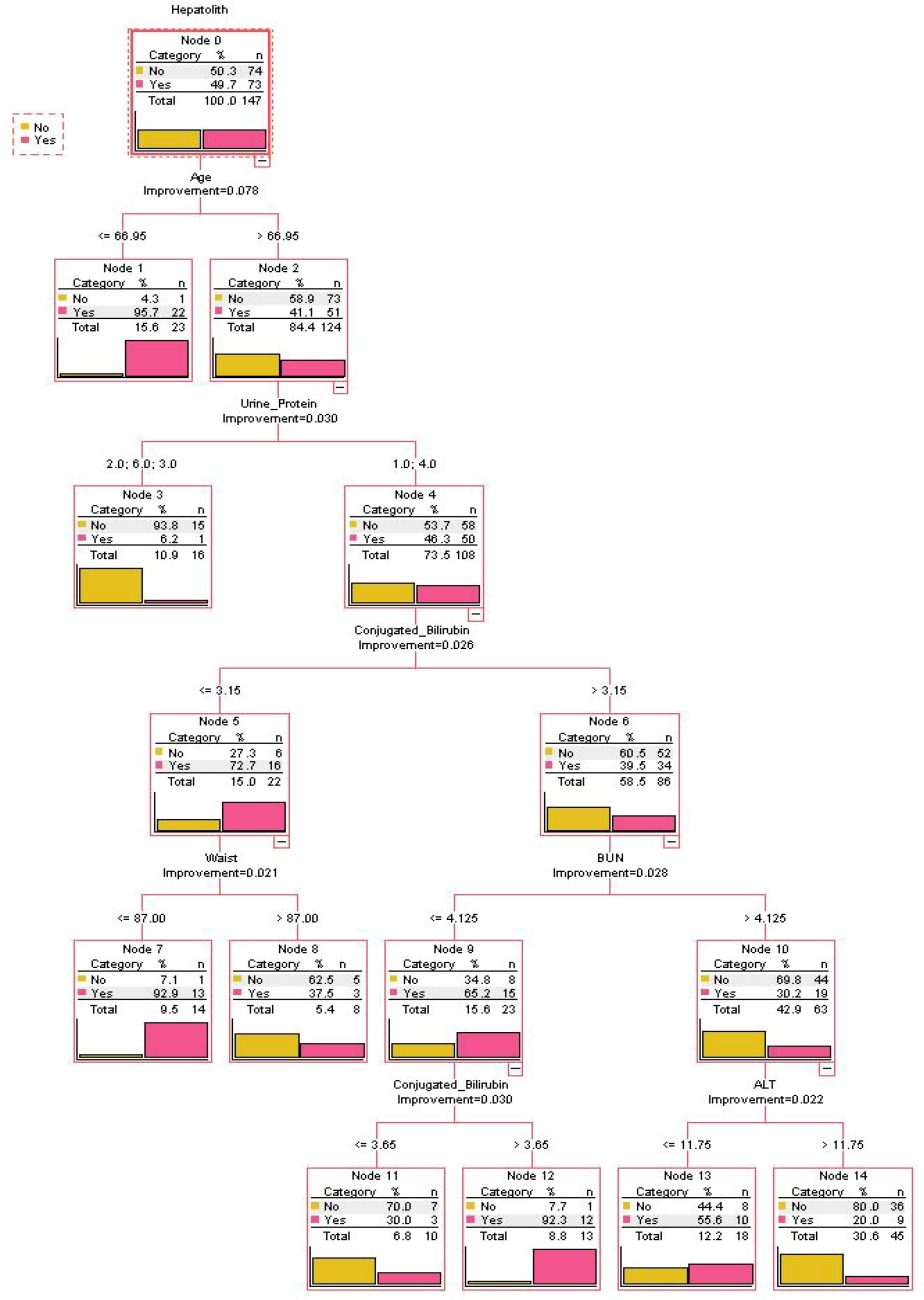
Decision tree model of factors influencing hepatolithiasis among pre-frail older adults for Group 2.

The Group 2 decision tree model had 15 nodes, eight terminal nodes, and a maximum tree depth of five. After 10-fold cross-validation, the model’s classification performance was almost identical to Group 1. The overall model accuracy was 81.6%, with prediction accuracy for non-hepatolithiasis and hepatolithiasis at 85.1% (63/74) and 78.1% (57/73), respectively. The model’s resubstitution risk was 0.184, and cross-validation risk was 0.456, very close to Group 1 results, both reflecting similar fitting and generalization characteristics under limited sample sizes. The gain table showed that node 1 (*n* = 23), node 7 (*n* = 14), and node 12 (*n* = 13) had the highest efficiency in identifying hepatolithiasis patients, with response rates of 95.7%, 92.9%, and 92.3%. The model’s primary split node was age (>66.95 years), followed sequentially by positive urine protein, conjugated bilirubin (>3.15 μmol/L), waist circumference (>87.00 cm), blood urea nitrogen (>4.12 mmol/L), conjugated bilirubin (>3.65 μmol/L), and ALT (>11.75 U/L). This indicates that this result, in the matched sample, further confirmed the core role of age (>66.95 years), positive urine protein, conjugated bilirubin (>3.15 μmol/L), waist circumference (>87.00 cm), blood urea nitrogen (>4.12 mmol/L), conjugated bilirubin (>3.65 μmol/L), and ALT (>11.75 U/L) in hepatolithiasis risk prediction (Figure 4).

**FIGURE 5:**
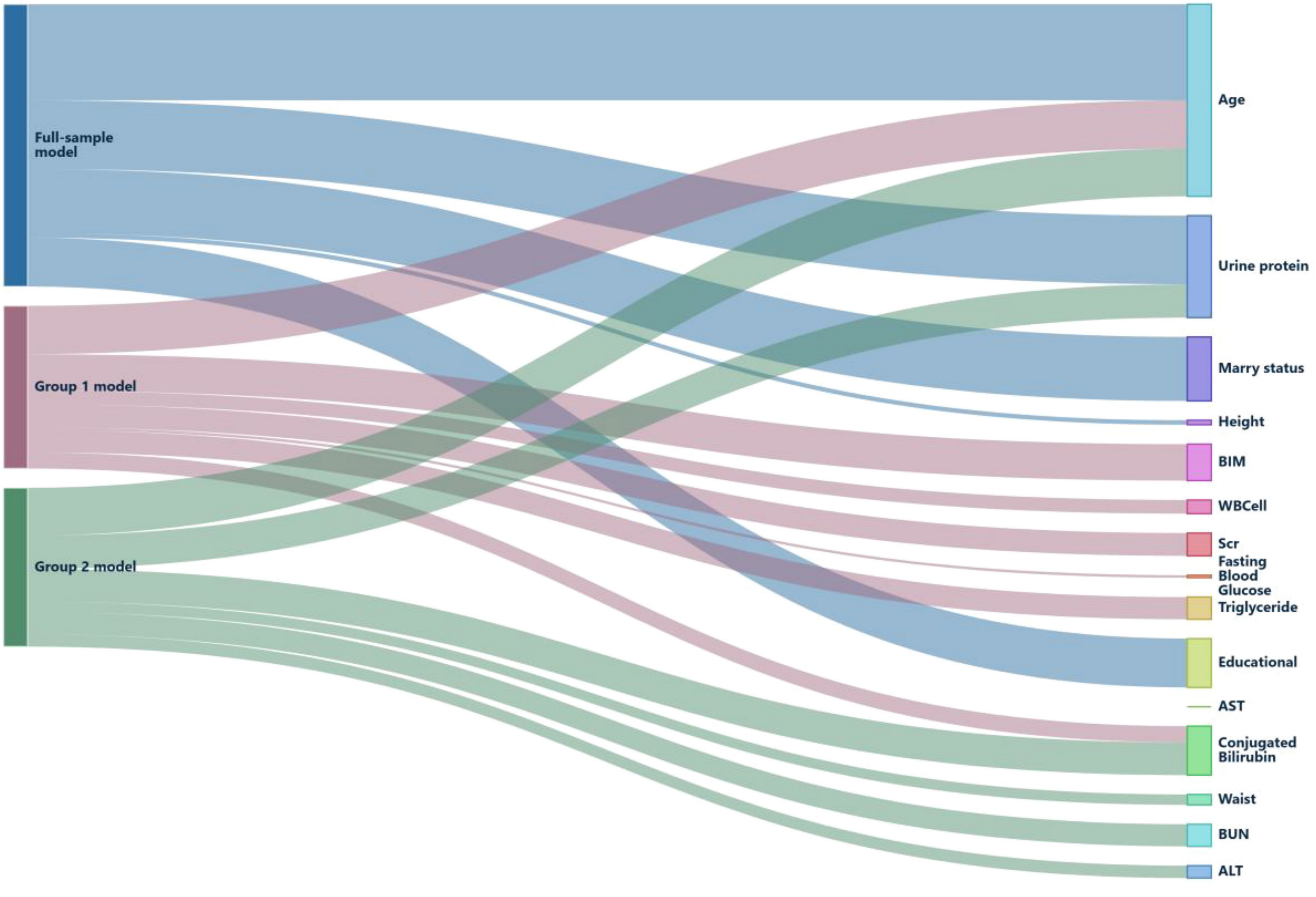
Sankey plots of risk factors for hepatolithiasis in three models.

The Sankey diagram visualizes key predictors across three models for hepatolithiasis risk in pre-frail older adults. Notably, age is consistently identified as a dominant risk factor in all models, highlighting its critical role in disease prediction. The full-sample model incorporates a comprehensive set of clinical and demographic predictors. These include age, height, body mass index (BMI), waist circumference, and educational level, alongside a wide array of laboratory measures such as urine protein, white blood cell count (WBCell), serum creatinine (Scr), fasting blood glucose, triglycerides, aspartate aminotransferase (AST), alanine aminotransferase (ALT), blood urea nitrogen (BUN), conjugated bilirubin, and a general blood indicator. Notably, the model is stratified into Group 1 and Group 2, suggesting that the effects of these variables are examined separately across two distinct subpopulations, possibly defined by a key clinical characteristic. This stratification allows for the detection of subgroup-specific associations that might be obscured in a pooled analysis. The inclusion of both renal (urine protein, Scr, BUN), hepatic (AST, ALT, bilirubin), metabolic (glucose, triglycerides, BMI, waist), and inflammatory (WBCell) markers reflects a holistic approach to risk prediction or outcome modeling. The presence of educational level as a social determinant further underscores the model’s effort to adjust for socioeconomic confounders. Overall, the full-sample model with its two parallel subgroup analyses provides a robust framework to evaluate whether predictor effects are homogeneous or vary meaningfully between groups, thereby enhancing the precision and clinical interpretability of the findings (Figure 5).

### 3.4 The AUC of three models

The Full Sample Model achieved an AUC of 0.795 (95% CI: 0.744–0.845) with 147 positive and 147 negative cases. In comparison, the Group 1 Model demonstrated higher discriminative performance, with an AUC of 0.914 (95% CI: 0.867–0.960) based on 74 positive and 73 negative cases. The Group 2 Model yielded an AUC of 0.877 (95% CI: 0.820–0.934) with 73 positive and 74 negative cases (Table 1).

**TABLE 1:** The area under the curve (AUC) of three models.

Models	Positive	Negative	AUC	95% CI
Group 1 model	74	73	0.914	0.867∼0.960
Group 2 model	73	74	0.877	0.820∼0.934
Full sample model	147	147	0.795	0.744∼0.845

**FIGURE 6:**
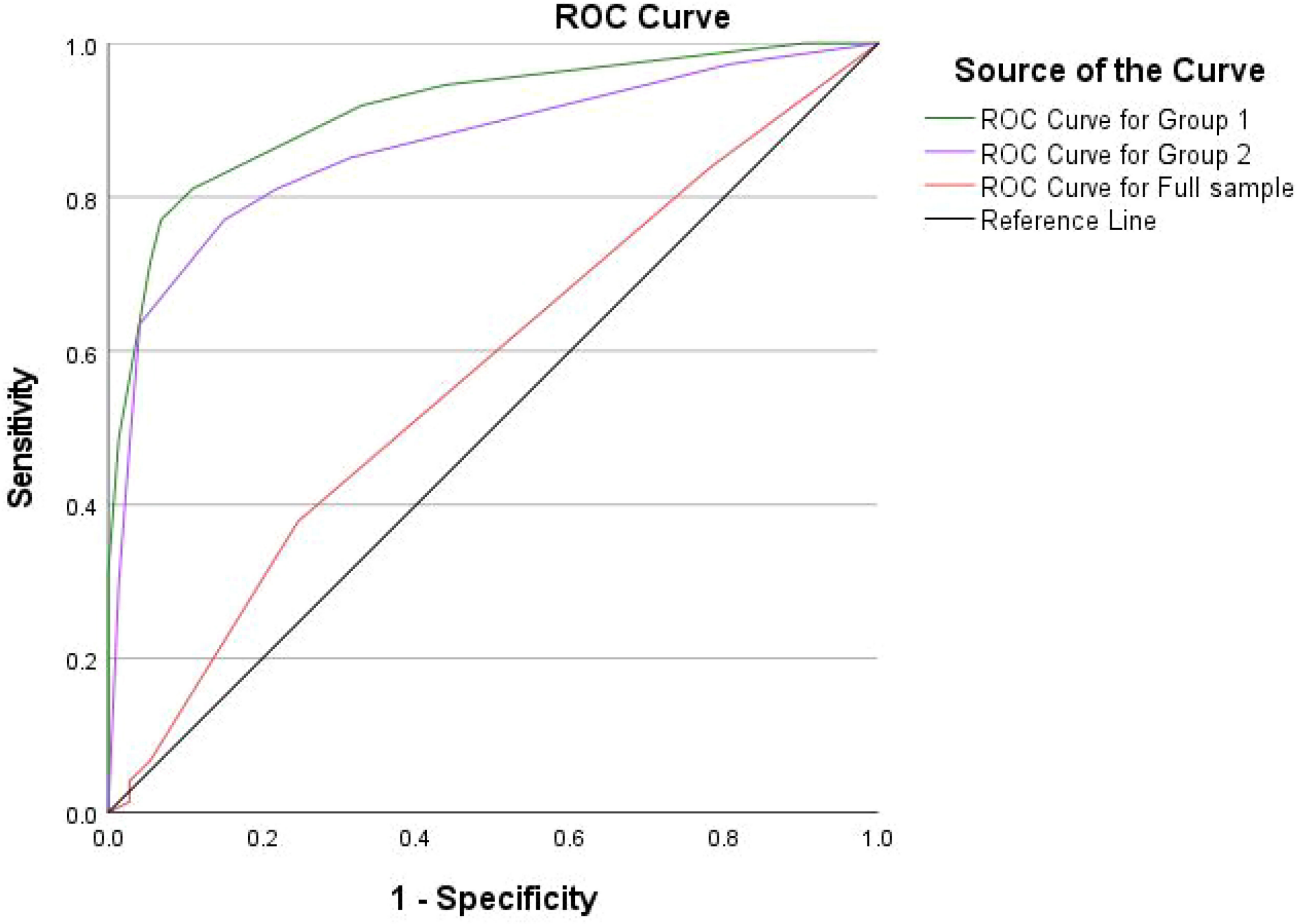
ROC and area under the curve (AUC) of three models.

The accompanying ROC curve plot visualizes the trade-off between sensitivity (true positive rate) and 1-specificity (false positive rate) for the three models. Consistent with the AUC values, the ROC curve for Group 1 Model shows the highest overall discriminative ability, followed by Group 2 Model, while the Full Sample Model exhibits the lowest performance. The reference line (diagonal) represents random guessing. The curves collectively indicate that the propensity score–matched subgroup models substantially outperformed the full sample model in classification accuracy, matched subgroup models achieved notably higher discriminative ability at the cost of increased overfitting risk (Figure 6).

## 4 Discussion

To our knowledge, this study represents one of the initial efforts to apply a decision tree to construct a risk prediction model for hepatolithiasis in pre-frail older adults. By innovatively comparing model results from the full sample and different subsets after PSM, it deeply explores model stability and the heterogeneity of risk factors, including age, blood lipids, blood glucose, liver function, kidney function, and some urine test indicators, and the model has good stability and repeatability.

### 4.1 Identification of core risk factors and model performance

The result of this study shows that the decision tree model can effectively integrate multiple clinical and demographic variables to construct an interpretable risk stratification path for hepatolithiasis. In the unmatched full-sample model, the model showed certain predictive ability and robustness, with an overall accuracy rate of 70.4%. However, it also suggested that there was a risk of overfitting, with a resubstitution risk of 0.296 and a cross-validation risk of 0.469. Key risk factors identified by the model include age > 66.85 years, strongly positive urine protein, marital status (married, widowed), height, and educational level. Among them, age is the primary dividing node, which is consistent with the epidemiological characteristics of hepatolithiasis as a chronic and progressive disease. This also means that the predicted age of onset for hepatolithiasis in Guangxi is younger than that shown in previous relevant literature, which indicated an age of over 66.85 years. Increasing age is an important risk factor for the accumulation of pathological changes in the biliary system (12). Strong urinary protein positivity may reflect the existing renal dysfunction or systemic micro-inflammatory state, which is potentially related to the possible systemic effects of chronic biliary infection and cholestasis (13, 14). The model results highlight that the development of hepatolithiasis is a complex process involving multiple factors.

### 4.2 Validation and expansion of model results after PSM

To control for potential confounding factors, the study employed PSM to obtain balanced control samples. The two independent decision tree models constructed after matching both demonstrated higher and consistent classification performance, with an overall accuracy rate of 81.0% and 81.6%, respectively. The cross-validation risk values for the two groups were 0.544 and 0.456, respectively, suggesting the need to be cautious of overfitting, which might be related to the reduced sample size after matching (15). The results of the two models were both consistent and divergent, providing a richer perspective for understanding the risk spectrum of hepatolithiasis.

### 4.3 Consistency found

Age was the primary and key risk stratification variable in both models with age > 66.45 years old in group 1; and age > 66.95 years old in group 2, once again confirmed that age is a core risk factor that cannot be ignored for hepatolithiasis. This result not only confirms the key role of age in the occurrence of hepatolithiasis, but is also highly consistent with the conclusions of many domestic and foreign studies (16, 17), highlighting its stable predictive value in the older adults population in the early stages of frailty. A survey study showed that the prevalence of hepatolithiasis increases significantly with age, and is more common in women, with age > 50 years old being a risk factor (18). This is mutually consistent with the result that age ≥ 66 years old is the primary segmentation node in the decision tree model of this study. In terms of pathophysiological mechanisms, aging is accompanied by decreased liver metabolic function, changes in bile composition, weakened bile duct peristalsis, and reduced immune clearance ability. These changes jointly promote cholestasis and stone formation (16). In addition, long-term accumulated biliary infection or mild inflammatory damage is more likely to develop into chronic bile duct lesions in the older adults, providing a pathological basis for stone formation (19). However, this study focused on a specific subgroup of older adults in the pre-frailty stage and further refined the risk connotations of age factors.

Compared with the general older adults population, although pre-frail individuals do not meet the frailty criteria, they already have early signs of decreased physiological reserve and multi-system functional decline. In this context, the physiological decline effects represented by age are amplified. This study found that among pre-frailty older adults people of the same age (≥66.45 years old), those with elevated chronic inflammatory markers had a significantly higher risk of hepatolithiasis. This suggests that age is not only a risk indicator in the time dimension, but may also accelerate the disease process in conjunction with other factors by superimposing physiological vulnerability in the pre-frailty state. In contrast, although some studies on the general adult population have confirmed that age is associated with hepatolithiasis (20), they have failed to reveal the amplification characteristics of this effect in pre-frailty groups, which highlights the clinical significance of this study’s model for stratified prediction for older adults high-risk subgroups. Notably, the decision tree model of this study showed extremely high predictive stability for the age node, which is in contrast to some studies that have explored modifiable risk factors (21, 22). Some studies have found that the impact of specific regions or dietary customs on hepatolithiasis may fluctuate with time or lifestyle changes. Low-protein and low-fat diets have a negative impact on bile excretion and promote cholestasis (18), but the risk effect of age remains consistent across different cohorts. This further confirms the status of age as a stable risk factor that cannot be ignored. In intervention practice, this means that for pre-frail older adults, age older than 66 years should be considered as one of the core basis for screening and preventive intervention for hepatolithiasis, even if other risk factors are not significant.

### 4.4 Difference and the risk of diversification path

The Group 1 model revealed risk pathways that were more related to metabolic and inflammatory conditions, including a higher body mass index, white blood cell count, fasting blood sugar, triglyceride levels, and lower serum creatinine. This suggests that in some pre-frailty older adults populations, the risk of hepatolithiasis may be closely related to components of metabolic syndrome and potential subclinical inflammation. The Group 2 model highlights the indicators directly related to the liver and gallbladder system, such as positive urine protein, elevated conjugated bilirubin, abnormal serum alanine aminotransferase, as well as a larger waist circumference and specific blood urea nitrogen levels. This path points more directly to the liver’s ability to process bilirubin, the possible role of biliary obstruction or hepatocyte damage, and the impact of central obesity as a metabolic risk factor. These risk factors point to metabolic abnormalities, liver function impairment, and renal function changes, highly consistent with the infection-metabolism-bile stasis multiple hypothesis of hepatolithiasis formation (23, 24), enhancing the biological plausibility of the models.

### 4.5 The instability of a single decision tree as a high-variance model

This study reveals that the specific risk variables for disease in the full sample model are inconsistent with those in the Group 1 and Group 2 models, which also exposes the instability of a single decision tree as a high-variance model, the main reasons are the randomness of the PSM process (25), which created two slightly different balanced samples, and the sensitivity of the greedy splitting algorithm of the decision tree to minor fluctuations in the data (26). When the predictive power of multiple related indicators such as serum alanine aminotransferase and serum aspartate aminotransferase, as well as waist circumference and body mass index, is similar, it is easy to select different representatives in different samples. This phenomenon suggests that if a single decision tree model is directly applied in practice, its generalizability may be limited.

### 4.6 Risk stratification and intervention grouping strategies

The decision tree model constructed in this study provides a visual tool for risk assessment of hepatolithiasis in pre-frail older adults people. The model effectively classified high-risk groups, with node 1 with a response rate of 100.0% and 95.7% in group 1 and group 2, respectively, are effectively isolated from the general population, which is helpful for clinical preliminary screening and risk stratification. The results of the study suggest that for the pre-frail older adults, the prevention and control of hepatolithiasis should not only focus on the hepatobiliary system itself, but also require comprehensive assessment, including routine monitoring of age-related changes in the biliary tract, attention to metabolic blood sugar, blood lipids, body mass index (27), and waist circumference (27) indicators, assessment of nutrition (24), white blood cell count inflammatory status, and examination of hepatobiliary and renal function markers such as bilirubin, transaminase, urinary protein, creatinine, and urea nitrogen. Integrated assessment of these factors may be better than a single indicator in identifying at-risk individuals. In practical applications, according to the combination of risk factors identified by the robust model, age combined with metabolic syndrome indicators or liver function abnormalities are used to carry out risk stratification and intervention grouping for pre-frail older adults people. The metabolic high-risk group should focus on lifestyle intervention related to blood sugar and blood lipid management; and the hepatobiliary high-risk group should strengthen liver function monitoring and regular ultrasound screening to achieve more targeted and precise health management.

## 5 Conclusion

Full-sample models reveal associations between hepatolithiasis risk and a wide range of physiological, metabolic, and social factors. In two sets of independent samples whose baseline characteristics were balanced through PSM, the constructed decision tree models were not only highly consistent in structure, but also showed almost the same classification performance and combination of key predictor variables. The core prediction structure of the decision tree model for predicting the risk of hepatolithiasis in pre-frail older adults, which was constructed in this study, is mainly based on age, blood lipids, blood glucose, liver function, kidney function, and some urine test indicators, and the model has good stability and repeatability, it provides a reliable data model basis for the subsequent formulation of precise intervention strategies for high-risk populations. This study has certain limitations. The sample size after PSM matching was still limited, which may be the reason for the overfitting of the model and also limits the universality of the identified risk factors in the wider population; although the decision tree model is highly interpretable, its stability may be inferior to ensemble learning models such as random forests; the study is a cross-sectional design, and the identified associations cannot confirm causality. Future research could combine PSM with ensemble learning algorithms such as random forests and gradient boosting trees to construct a more precise risk precision model, and ensemble methods can provide a more stable and reliable ranking of variable importance by aggregating the results of multiple trees, resulting in a consensus set of core risk factors.

## Data Availability

The raw data supporting the conclusions of this article will be made available by the authors, without undue reservation.
